# Using mobile money data and call detail records to explore the risks of urban migration in Tanzania

**DOI:** 10.1140/epjds/s13688-022-00340-y

**Published:** 2022-05-08

**Authors:** Rosa Lavelle-Hill, John Harvey, Gavin Smith, Anjali Mazumder, Madeleine Ellis, Kelefa Mwantimwa, James Goulding

**Affiliations:** 1 University of Tübingen, Tübingen, Germany; 2 The Alan Turing Institute, London, UK; 3 The University of Nottingham, Nottingham, UK; 4 University of Dar es Salaam, Dar es Salaam, Tanzania

**Keywords:** Mobile money, Machine learning, Migration, Call detail records, Exploitation, Tanzania, Vulnerability

## Abstract

Understanding what factors predict whether an urban migrant will end up in a deprived neighbourhood or not could help prevent the exploitation of vulnerable individuals. This study leveraged pseudonymized mobile money interactions combined with cell phone data to shed light on urban migration patterns and deprivation in Tanzania. Call detail records were used to identify individuals who migrated to Dar es Salaam, Tanzania’s largest city. A street survey of the city’s subwards was used to determine which individuals moved to more deprived areas. *t*-tests showed that people who settled in poorer neighbourhoods had less money coming into their mobile money account after they moved, but not before. A machine learning approach was then utilized to predict which migrants will move to poorer areas of the city, making them arguably more vulnerable to poverty, unemployment and exploitation. Features indicating the strength and location of people’s social connections in Dar es Salaam before they moved (‘pull factors’) were found to be most predictive, more so than traditional ‘push factors’ such as proxies for poverty in the migrant’s source region.

**Supplementary Information:**

The online version contains supplementary material available at 10.1140/epjds/s13688-022-00340-y.

## Introduction

Urban migration can hold both potential benefits and risks to individuals, societies and economies: on the one hand cities can present increased work opportunities, higher paid jobs, and a greater capacity to provide for families, even from afar. However, such urban settings can also be unstable, with a higher cost of living, leaving individuals potentially isolated and vulnerable [1]. At a national level, when urban migration rates exceed investment in job creation activities, the result is upward pressure on job competition leading to increased unemployment and a risk of exploitation—with some migrants being left in poverty and without support [2]. Yet the percentage of the world’s population living in urban areas is expected to increase from 55% in 2018 to 60% in 2030 [3], with most of the world’s fastest growing cities being in Asia and Africa. Between 2018 and 2050 the urban population of Africa is projected to triple; and that of Asia is expected to increase by 61%. Despite this there remains limited understanding of the how such fast paced migration is impacting communities on the ground.

Measurement remains the key challenge, with surveys such as national censuses (which typically run every 10 years) becoming rapidly out-of-date [4]. This raises serious logistical difficulties regarding how to locate, capture and study the impacts of domestic urban migration—particularly in lower-income countries and at sub-national levels. Mobile phones provide a potential solution here. Due to historically under-developed land-line infrastructures, mobile devices have become ubiquitous across Africa and Asia, even in poorer or geographically isolated regions [5]. Billions of people now carry such devices, supporting the analysis of broad movement patterns through time and space. The use of personal data rightly raises important ethical considerations and requirements [6, 7]; yet if handled correctly, safely and respectfully, call records and other digital traces have the potential to reveal otherwise unavailable insights about short term, seasonal, and quickly changing domestic migration patterns. Such information combined with computational modelling approaches can underpin the inspection of different sustainable development goals at more fine-grained resolutions [8–10].

A contributor to the increase of urban migration in Africa and Asia is thought to be the uptake of mobile money [11]; mobile money allows migrants to quickly and securely transfer funds home to their families without reliance on formal banking infrastructures (which are often unavailable to them). Over the last 10 years, use of mobile money has consequently become common-place in many lower-income countries, with uptake expanding further during the COVID-19 pandemic [12]. Use of the technology has grown particularly quickly in East Africa [12], and in 2017 only 38% of adult males and 27% of adult females held a bank account with a formal financial institution [13]. Registered mobile money accounts in Africa grew a futher 12% to 562 million in 2020, with monthly active accounts at 161 million (an 18% increase), and total transactions hitting 27.5 billion (up 15%) [14]. In Kenya the money transferred through *M-Pesa* service alone increased from 30% of the country’s GDP as of 2011, to 85% by 2016 [15].

Despite a large body of literature documenting the benefits to communities of mobile money remittances [16–19], less has been recorded about the welfare and hardships faced by the individuals who migrate to urban areas in search of employment. Qualitative literature suggests that new migrants represent a particularly vulnerable community: a lack of opportunities in particular neighbourhoods increases the risk of poverty, exploitation, and even indentured labour and trafficking [2, 20]. Yet migrants are not a homogeneous group. The exact mechanisms by which some individuals are left vulnerable have not been quantitatively examined, nor the characteristics of those most likely to end up in high-poverty, high-unemployment neighbourhoods, where social and economic impacts are most deleterious. Whilst slums, and the vast poorly serviced informal settlements that now make up the majority of East African cities, have been linked to increased exploitation [9], digital data traces such as those from cell phones and mobile money interactions offer the potential to quantitatively address this, and shed new light on these issues.

In this research we use Tanzania as a case study to explore the associated behaviours and predictors of migration to poorer urban areas within a low-income country. By leveraging pseudonymized mobile money transactions and cell phone data from a commercial mobile network provider, combined with survey data, we examine domestic migration patterns in Tanzania, and model corresponding vulnerabilities that can exist. We use call records to detect permanent/semi-permanent migration to Dar es Salaam, Tanzania’s largest city. Utilising an extensive street survey of Dar es Salaam’s 452 sub-wards, we are able to determine whether each person has migrated to a more vulnerable (higher poverty/higher unemployment) or a more affluent subward. Features derived from the call data and mobile money interactions, as well as open source socio-demographics information on the region an individual has migrated from, are used to generate predictive models; variable importance analysis is then used to interrogate the resulting model, to consider the characteristics of higher-risk urban migration.

With individuals migrating to deprived neighbourhoods hypothesised as being those most exposed to risk [21], the goals of this study are twofold: (1) Understand the differences, visible in digital traces, between migrating to a poorer urban area compared to a more affluent area by statistically comparing social and economic measures *after* individuals have moved. It is hypothesised that individuals migrating to poorer areas will have less money coming into their account (potentially indicating a financial vulnerability). Other indicators of risk for these migrants may include reduced social connections or contacts. (2) Uncover which underlying factors best *predict* migration to an economically deprived area, in the hope of supporting sustainable development assessment and informing future intervention strategies.

The following section further expands relevant literature about mobile money, cell phone data, and migration. We then present our methods, explaining how urban migration can be detected using call detail records, how we engineer and select relevant features, and our modelling method. The results section follows, outlining findings from two different types of analyses (1) using statistical tests to compare the social and economic differences between migrants who moved to poorer compared to richer areas of Dar es Salaam, and (2) the accuracy and interpretation of a prediction model, built to predict which migrants will end up in the more deprived areas of Dar es Salaam. In the final section, we discuss our results and their limitations, as well as suggesting avenues for future investigation.

## Related work

### Mobile money and migration

Mobile money is now shaping the economic and cultural landscape in many African and Asian countries, promoting migration and urban occupations [16]. In Bangladesh, mobile money increased the value of remittances by 28% and the migration rate by 35% [18]. Households in Bangladesh that had urban migrants and actively used mobile money saved 296% more than nonusers [18]. It was additionally found that mobile money increased daily per capita consumption by 8% and reduced the extreme poverty index by 42% when urban migrants remitted income back to their household in rural Bangladesh [18]. In Northern Uganda, mobile money was shown to increase food security by 45% for households that lived far away from bank branches [17].

Research has also shown that mobile money can help protect against the effects of negative shocks, such as flooding, due to increased capacity to receive financial support. For example, in Kenya remittances increased households’ annual income by 3-4% following a negative shock; and mobile money users saw no change in their consumption level, compared to nonusers who showed a 7% decrease [19]. In Mozambique, mobile money was shown to increase consumption expenditure by 44% after a flood shock [22]. For low-lying countries such as Bangladesh, programmes such as forecast-based financing which use weather forecasts to trigger early actions such as cash transfers can help reduce the impact of a natural disaster. Increased resilience to negative shocks has the potential to make *reactive* or the *forced* migration of whole families less common; instead increasing the reliance on remittance payments from just a few individuals (most commonly young males) who have migrated to a city for work.

Tanzania was one of the earliest adopters of mobile money, and since its launch in 2008 adoption rates have been high [23]. In 2015, almost a third of active mobile money accounts in East Africa were in Tanzania [24]. In 2016 approximately half of the total population were mobile subscribers, over half the adult population were mobile internet subscribers, and it was estimated that 32% of adults in Tanzania had a mobile money account [23]. Research has also shown that the reach of mobile money in Tanzania has good representation across multiple populations, and crucially those living in rural areas, the unbanked, and those that earn less than $2 per day [9, 25].

### Urban migration in Tanzania

Tanzania’s urbanisation has accelerated rapidly, and at a rate higher than the average for Africa [26]. Dar es Salaam, is by far Tanzania’s largest city, reportedly three times the size of it’s next biggest city, Mwanza [27]. It is not only one of the most populated in Africa, but also one of the fastest growing [27, 28]. As a result of this rapid growth, over 70% of Dar es Salaam’s population live in unplanned urban sprawl and informal settlements, often without adequate housing, safe drinking water, or affordable sanitation [26, 27, 29]. The region in which Dar es Salaam is situated is one of the more affluent regions in Tanzania; yet it is also characterized by a far higher unemployment rate than the rest of the country and an increasing Gini-index (a measure of the distribution of income across a population) [26], reflecting the growing disparity between its rich and poor inhabitants.

A key driver of domestic migration to urban areas in Tanzania is low rural income, most commonly in agricultural sectors. After migrating to urban areas, one study found that 63.4% of rural–urban migrants in Tanzania were engaged in petty businesses in the informal economy [30]. Yet the higher cost of doing business in Dar es Salaam means that very few new businesses survive [26]. 60% of rural–urban migrants in Tanzania were able to save and send remittances to their place of origin [30]. But what about the 40% who were not able to send money home? Who are these ‘worse-off’ individuals and what are the effects of migration upon them? Such questions are extremely difficult to address via direct surveying, a method that often misses those who are most vulnerable [31] and most at risk from exploitation (such as human trafficking and forced labour [2]). Even for the migrants who are able to save money, getting to a point of stability can take time, with individuals encountering a plethora of problems in the meantime, such as living in poverty, poor health, or unsafe conditions [30].

An influx of migrants to urban centres can also place increased strain on a city’s ability to cope with citizen needs. As a result, many migrants can be left without access to social support or afford adequate housing. In such regions, migration has been viewed by the local population as detrimental to society, contributing to shortages of housing, infrastructure, and services [32] and subsequently causing migrants to be viewed unfavourably and discriminated against. These factors can leave urban migrants more vulnerable to deprivation, homelessness, disease and violence [21, 33]. Migrant women, especially those who are undocumented, are also more likely to experience labour market exploitation and are at greater risk of kidnap or trafficking [34]. Yet little is known about what factors might help to inform support services as to *which* migrants will end up in vulnerable circumstances—whether that be poverty, unsafe and unsanitary conditions, or exploitation.

### Digital trace data from mobile phones

People migrate to a large city like Dar es Salaam for many reasons: to study at university, to take up a job offer/transfer, seasonal work such as tourism, for marriage, as a result of a negative shock, or because they are struggling financially and are looking for a better life [35–37]. With the fast changing urban landscape in many African and Asian countries, collecting data using surveys such as the national census can prove difficult logistically, are expensive, and can yield inaccurate or out-dated results [4, 38]. Most censuses occur every ten years, have low granularity, and the validity of the information is rapidly outdated [39, 40]. In particular, shorter term migration patterns or seasonal migration is not captured, both of which are highly prevalent in developing countries [4]. Moreover, censuses are typically biased toward documented citizens [41]; and in countries where illiteracy is relatively high, written/postal surveys risk excluding and marginalising a vulnerable sub-population [31].

Over the years, several migration studies have identified the scarcity of reliable data available for quantitative analysis as a challenge to be overcome, particularly in developing countries [39, 42, 43]. Novel data types such as digital traces have been proposed as proxies for traditional census data; assisting in analysis of urban migration in countries where such surveying is challenging [44]. As previously detailed, mobile phones are now ubiquitous in Africa and Asia, with the billions of people carrying such devices. The data produced from peoples interactions with mobile phones reflects real behaviour (rather than self-reported behaviour, as in censuses and other surveys). As such, data logs from network services represent a promising route to analysis of migratory behaviour both geographically and temporally, with a range of studies utilizing such geo-located data to study mobility patterns [40, 45–54].

Several studies have used mobile phone data to study mobility and migration patterns in developing countries specifically [4, 55–57]. Yet despite multi-modal data from different contexts improving prediction accuracy [58], no research, to the best of our knowledge, has attempted to use this data combined with mobile money transactions to assess the characteristics and potential social and economic consequences of urban migration to the migrant themselves; nor examined the factors that implicate the deprivation level of *where* people migrate to within urban settings. While academic research on mobile money and migration has previously focused on the rural communities left behind, one prior work [59] has shown that social networks in a *destination* location can strongly impact the success of a migration. This study expands this isolated research, examining not only how often, but for whom urban migration is likely beneficial.

## Methods

### The data

This work is underpinned by two key datasets:

1. Pseudonymized transactional data shared by a leading Tanzanian mobile network operator, comprising of (i) mobile phone call data records; and (ii) mobile financial services or *mobile money* data which can be linked to the call records. Using these call records, migrants to Dar es Salaam were identified. From both the call and mobile money data, associated features were engineered and used to (1) measure the differences in mobile money and call activity between those moving to a poorer versus richer subward, and (2) predict the likelihood a given individual would migrate to an area of deprivation in Dar es Salaam.
2. An extensive street survey administered by the authors to provide ground-truth measurements for deprivation levels across subwards in Dar es Salaam. This data was used, in combination with the call records, to label whether a migrant moved to a poorer or more affluent part of the city—the dependent variable in the prediction model.

#### Call records and mobile money transactions

The call data consisted of logs every time someone received or made a call in 2014. This data allowed us to track movement patterns of individuals over time. Call detail records represent the majority of mobile phone activity in Tanzania. Voice calls make up 50% of revenue from mobile devices in Tanzania, compared to just 10% for both data and SMS (the remaining 30% is from mobile financial services) [60]. The call data was pseudonymized before being received, so that individuals were only linkable by a unique identifier. Using these, the call data was able to be attached to mobile financial services data, also from the same commercial provider. Mobile money data consisted of a log every time a customer of the service sent or received money, or checked their balance. The data used in this study covered a total of 800,157,047 call events, and 48,435,309 transactions from 27,625 mobile phone subscribers in the Dar es Salaam region over the year 2014.^1^ To help provide better contextual understanding of the data and findings, the project engaged with local experts on mobile money and migration in Tanzania, and Dar es Salaam more specifically.

#### Street survey

Dar es Salaam is divided into 452 administrative areas referred to as *subwards*, which are the lowest formal level of administrative division in the city. The ‘street survey’ data collected in Dar es Salaam consists of these subwards ranked by affluence. Rankings were assigned from 75,078 comparative judgements made by 224 local participants, whom we refer to henceforth as judges.

To collect the data, a participatory approach was used to quantify knowledge and opinions of local residents on the ground in Dar es Salaam. To carry out the judgements, a web interface was designed so that judges could be shown images of pairs of subwards and asked to compare the affluence. At the start of the survey, judges were asked to identify areas of the city they were familiar with. Then, during the judging process, judges had the option to indicate either (i) which of the two subwards they felt was more affluent, (ii) that the subwards were roughly equal in affluence, or (iii) that they were unfamiliar with at least one of the two subwards.^2^ Pairs of subwards for each judge were chosen uniformly at random from the list of all possible pairs of subwards which the judge was familiar with. For further information on the methods used for obtaining the ranks from comparative judgements see [61].

Judges were recruited through word of mouth by students at local universities, NGOs, and via a local taxi driver association. The rationale was to find judges that were citizens of Dar es Salaam with a wide working knowledge of the city’s different subwards. Data was collected *in situ* over two weeks in August 2018 via 17 data collection sessions each lasting two hours. At the start of each session, judges received a 15 minute training session in English and Swahili, and accompanying written instructions were also provided. Ethical approval for the study and its data collection process was obtained from the Nottingham University Business School ethical review committee, application reference No. 201819072.

### Identifying migration to Dar es Salaam

Before we could utilize the large call and mobile money datasets for our analysis, the data required some cleaning and labelling. The end goal was to label anonymous individuals in the data who we could be fairly certain, given their geo-located and timestamped call data, had migrated to Dar es Salaam in the time frame we were interested in. To make the labelling process more efficient, we first cleaned the data to remove individuals we were certain we were not interested in including in our sample (due to poor quality data, or their data not fitting our definition of migration) using some filtering rules. These rules were carefully constructed after interrogating the data, and were designed to prioritize data quality over data quantity. For example, if an individual had too few mobile interactions either before or after migrating, we did not want to include them in our final sample, as the features engineered (including pinpointing the subward they migrated to) would be inaccurate and produce unreliable indicators of the individual’s actual behaviour or circumstances.

Specifically, we were interested in identifying anonymous individuals, with good quality data, who had moved permanently or semi-permanently to Dar es Salaam in the middle third of the year, from anywhere outside of the Dar region (but still within Tanzania). To eliminate individuals who obviously did not fit this definition, we first mined the call detail records using the following rules:

1. To ensure enough data coverage and temporal stability, anonymized individuals needed to have made or received >10 calls in both the first 6 months of the year and the last 6 months of the year.
2. In the first half of the year <30% of the individual’s calls should have been made/received within Dar es Salaam, and in the last 6 months of the year >70% calls had to have been made/received from within Dar es Salaam. This was to eliminate individuals who might have moved at the start or the end of the year, and thus have inadequate data for a fair before and after moving comparison.
3. To estimate the potential move date, the date of the individual’s first stay of more than 7 consecutive days in Dar es Salaam was taken, using the cell tower location attached to the call records. This prevented us from capturing people who commuted to Dar es Salaam for work, or who were only visiting for a short stay.
4. After the move date was estimated, the individual had to have made >75% of their calls (from their move date to the end of the year) in Dar es Salaam. This was to remove people from the sample who are only visiting Dar es Salaam on a short-term/temporary basis.
5. Finally, to ensure the potential migrants had sufficient mobile money data for us to analyse and engineer features from, individuals had to have at least 10 or more mobile money transaction logs (triggered by either receiving or sending money, or checking their balance).

**Figure 1:**
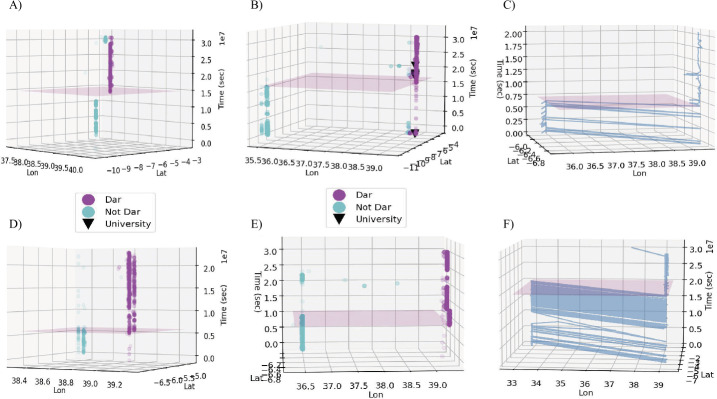
Examples of the visualisations of call detail records used by human labellers to determine whether it could be accurately estimated whether and when someone migrated to Dar es Salaam. The estimated move date is depicted by the pink hyper-plane. Each graph is a different individual, and each translucent dot represents a mobile phone call. The black triangle denotes a call made from the University of Dar es Salaam’s campus. All examples were labelled as correctly identifying migration to Dar es Salaam, with the exception of **F** which was deemed to have too large of an overlap due to what looks like regular commuting before migrating

Using these filters, from the 27,625 individuals, a sample of 1214 potential migrants to Dar es Salaam was extracted, along with estimated move dates. Using 3D plots to visually interrogate the individual’s movement patterns, each person’s data in the sample was then labelled by human subjects on whether or not the person’s data fitted our definition of migration, and whether the estimated move date was valid or not. Discussions were had prior to labelling as to what constituted a valid case of migration and move date, and what did not. Examples of the two types of 3D plots used in this stage (which were rotatable for the labeller in the provided interface) can be viewed in Fig. 1. Whether the individual had used a cell tower on the subward corresponding to the University of Dar es Salaam’s campus was visualized to help interpret mobility patterns which may be linked to university students. All graphs in Fig. 1 except graph A show individuals that visited Dar es Salaam prior to migration, a phenomena found to be a common occurrence in the data for people who didn’t live too far away. Graph B shows an exemplar migrant affiliated with the University of Dar es Salaam. Graph D illustrates how both a broad work and a home location might be identified in the data.

Note that, in addition to the historical nature of the data, to ensure differential privacy was strictly observed we restricted location resolution in Dar es Salaam to one of the 452 subwards (with subwards having an average of approximately 15,000 inhabitants each). Nonetheless broad movement patterns could still be labelled, with Graph E showing an example of someone who visited their previous home region for an extended period after moving to Dar es Salaam. Graph F suggests the behaviour of a person who was commuting regularly to Dar es Salaam before moving there permanently (but was deemed too large of an overlap to be considered in our sample). If a single move date could not be confidently determined from the visualisations then the individual was excluded from the sample. In total 848 of 1214 instances were labelled with a move date thought to correctly depict when someone had migrated to a Dar es Salaam subward on a permanent/semi-permanent basis.^3^ This subsample of 848 urban migrants was used for the remainder of the analysis. While this sample is relatively small (due to the limitations imposed by the data coverage and our working definition of migration), this work provides a first look at a well-defined subgroup of urban migrants to Dar es Salaam, that is expected to be much larger in practice. The data challenges are considered in more detail in the Discussion.

### Engineering the dependent variable

Classification of whether an individual migrated to a deprived or more affluent area within the city, was engineered using the street survey of Dar es Salaam in combination with the call records. We first estimated where in Dar es Salaam we thought a person’s new ‘home subward’ was using call data, and then linked this to the affluence rankings of the subwards, as derived from our surveyed comparative judgement ground truths (see [61]).^4^

**Figure 2:**
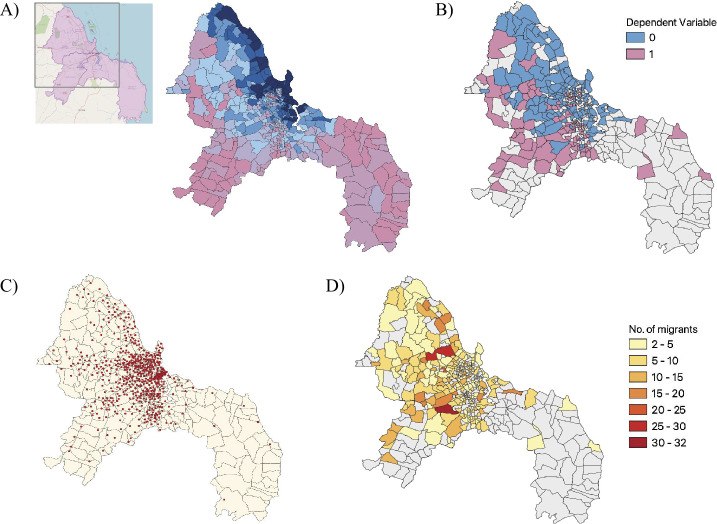
Maps of Dar es Salaam. **A**) The subwards’ (continuous) level of affluence (blue) to deprivation (pink). **B**) The spatial distribution of the dependent variable—the binary categorisation of the subwards migrants moved to (affluent = 0, deprived = 1). Grey areas indicate no one in the sample migrated there. **C**) Cell tower coverage across Dar es Salaam. Temeke, the most South Easterly region, was later removed due to poor cell tower coverage. **D**) The number of migrants in the sample who migrated to each subward

The area someone moved to in Dar es Salaam was estimated using the call records and cell tower location data. First, each individual’s ‘home tower’, the cell tower a person made the most calls with at night time (specified as between the hours of 8 pm and 8 am)^5^ was identified. Then, the destination subward was simply defined as the subward in which the home tower geographically lay within. 602 towers provide network coverage across the 452 subwards in Dar es Salaam. Figure 2(c), illustrates the coverage of cell towers across the subwards. To deduce a final binary outcome variable, we then calculated whether someone moved to a poorer area (50%^6^ most deprived subwards) or a more affluent area (50% least deprived subwards). Migrants whose destination sub-ward was in the district of Temeke (the southern most region of Dar es Salaam) were removed due to reduced network coverage (see Fig. 2(c)), historically different network governance, as well as the less urban nature of the district. This reduced our modelling sample to 630 individuals of which 230 (36.5%) moved to more deprived areas, and 400 (63.5%) moved to the more affluent areas. The spatial distribution of the dependent variable can be viewed in Fig. 2(b), along with a heatmap of how the migrants were distributed across the city (Fig. 2(d)).

### Engineering the independent variables

As potential indicators of vulnerable migration, a total of 110 candidate features (*K*) were engineered from aggregating cell phone data, mobile money data, and open source data sources [29]. A full list of the candidate features, which analysis they were used for, and their descriptions can be found in Additional file 1. Different versions of features were engineered via: (1) using only the data before the person moved ($K = 93$); and (2) using only data after they had moved ($K = 17$). These two sets of features were used in separate analyses: data from *after* moving to statistically analyse the social and economic differences between those who moved to a poorer versus richer subward; and data from *before* moving to predict whether an individual would migrate to a poorer or more affluent area of Dar es Salaam. As part of the modelling analysis, feature selection methods (outlined in more detail below) were applied to reduce the number of candidate features.

Features from the cell phone data were engineered to reflect social connectedness, as well as existing ties and connectivity with Dar es Salaam (‘pull factors’). Examples of these features include: the entropy of the numbers called; average calling distance; the percentage of calls to made to Dar es Salaam (before moving); the affluence of the area most commonly called in Dar es Salaam (before moving); and whether the individual had visited Dar es Salaam prior to moving there. Entropy features were calculated using Shannon entropy (H(X)) with a the natural logarithm:

$$ H(X) = -\sum_{i=1}^{n}p(x_{i})*\ln(x_{i}). $$

Features from the mobile money data were engineered as potential proxies for an individual’s financial situation. Examples of these features include: whether the person had a mobile money account before moving to Dar es Salaam; their mean mobile money account balance, the average amount of money paid into the account per day; the average amount of money spent per day; amount paid out in bills; and the amount sent/received from person-to-person transfers.

Features about the region a migrating individual originated from were extracted from open source data [29]. These features reflected proxies as to the level of deprivation an individual was migrating from, representing the strength of migratory ‘push factors’. These regional variables covered a wide range of domains including: human development indices, poverty, education, gender inequality, female representation in parliament, health, and population demographics [29]. Once aggregate features for each anonymized migrant had been constructed and attached to a deprivation level of the subward they migrated to, all other call and mobile money data were expunged from the study.

### Modelling

In order to reduce the effects of excessive multi-colinearity and the curse of dimensionality, expected to be present due to the types of features constructed, two pre-processing steps were undertaken on the 93 features engineered for modelling (on data before individuals migrated). Addressing these issues, input features which were highly correlated ($\text{Pearson }r~> 0.85$) were eliminated or averaged. Subsequently, features which had a Pearson correlation with the dependent variable of less than a fixed value were removed. We note that such an approach risks removing features that, while do not have any direct relationship with the output feature contribute do in fact have a relationship when considered in combination with other features. Acknowledging this we varied the Pearson correlation cut-off when considering the correlation between the input features and dependent variable from 0.05 to 0.07 (effectively as an additional meta-parameter for all models in the machine learning pipeline described below). As varying this parameter lead to no discernible decrease in predictive performance (demonstrating the limited utility of the features dropped with the higher threshold) in the remainder of this paper, for clarity, we consider models only where the cut-off was set to 0.07. After applying this pre-filter, the number of modelling candidate features was reduced from 93 to 15 features engineered on data prior to an individual’s migration to Dar es Salaam. A list of these features can be found in Additional file 1.

A machine learning pipeline was then built to predict whether someone ends up in a poorer or more affluent subward. The pipeline consisted of imputation of missing data (see Table 1 for missing data information) in the IVs using multivariate imputation [66], data scaling, recursive feature elimination,^7^ and the training of a classification model. Three classes of classification algorithm were evaluated: logistic regression, decision trees, and random forests—all chosen for their interpretable variable importance outputs. Hyper-parameters used within the pipeline were selected using 10-fold cross validation on an 80% subsample of the data ($N = 504$) with the cross-validation procedure splitting this sample repeatedly into training and validation sets. The models were then re-fit on the full 80% sample based on the selected meta-parameters. The remaining 20% of the data ($N = 126$) was used as an unseen test set to evaluate the generalised predictive performance.

**Table 1:** Modelling features which had missing data and were imputed using multivariate imputation

Feature	% missing
Money sent before migrating (normed)	6.13
Deprivation of ward most phoned	6.01
Deprivation of subward most phoned	6.01
Percentage of calls to Dar es Salaam	5.78
Call entropy before migrating	5.78
Home region: Education level	3.07
Home region: Measles immunization	3.07
Home region: Antenatal visits	3.07
Home region: Parliament female:male ratio	0.47
Home region: Population	0.47

## Results

### Descriptives

**Figure 3:**
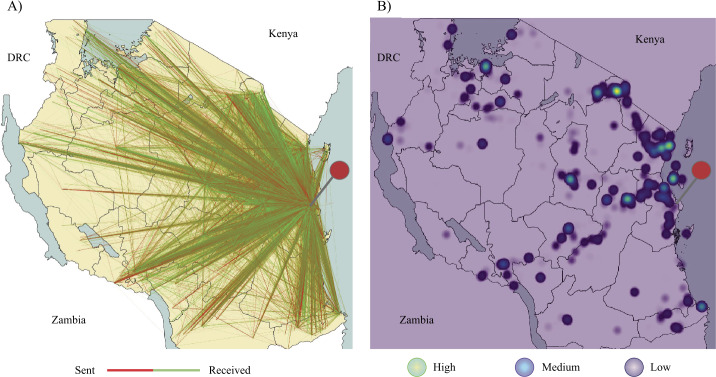
Descriptive illustrations of **A**) the person to person transactions in the full mobile money dataset, and **B**) the density of call data outside of Dar es Salaam for the modelling sample, a proxy for where people migrated from. The red pin indicates the city of Dar es Salaam. DRC = Democratic Republic of the Congo

Within the sample of 848 migrants, the median number of calls people made before migrating to Dar es Salaam was 384 ($\mathrm{SD} = 687$), and after moving was 608 ($\mathrm{SD} = 708$). Use of mobile money also increased following migration, with transactions prior (Mean = 52, $\mathrm{SD} = 58$) markedly higher than before (Mean = 43, $\mathrm{SD} = 61$)—likely due to the more pervasive mobile money infrastructure available within Dar es Salaam. Figure 3(a), showing all person-to-person transactions in the full data set, illustrates Dar es Salaam’s (marked by the red pin) role at the centre of the mobile money network in Tanzania. Figure 3(b) shows all calls outside of Dar es Salaam in the modelling sample, a proxy for where individuals migrated from. People migrate to Dar es Salaam from across Tanzania, although we observed particular hotspots in the urban centres of Dodoma, Arushka, Mwanza, Tanga, Zanzibar, and Mtwara. When the urban/rural population of individual home wards was analysed, we found that roughly the same amount of people were migrating to Dar es Salaam from predominately urban (53%) as predominately rural (47%) wards. Therefore, our analysis of migrants to Dar es Salaam more or less equally captures rural–urban and urban–urban domestic migration patterns.

### Statistical tests

**Figure 4:**
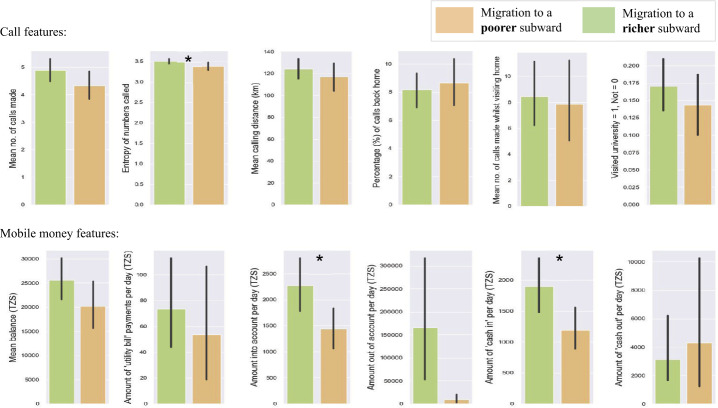
Differences in call behaviour and mobile money patterns after migrating between those who moved to a poorer neighbourhood and those who migrated to a more affluent area. $\boldsymbol{*} = p < 0.05$. Error bars denote the 95% confidence intervals

**Figure 5:**
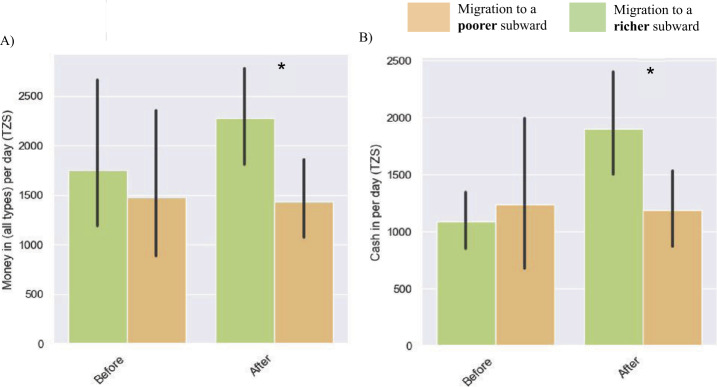
Differences in **A**) money in (all types) and **B**) cash paid in to mobile money accounts before and after migrating between those who moved to a more deprived neighbourhood and those who migrated to a more affluent area. $\boldsymbol{*} = p < 0.05$. Error bars denote the 95% confidence intervals

To analyse how the migrants to poorer and richer subwards’ call and mobile money patterns differed after moving, statistical analyses were performed. *t*-tests were conducted on six call features, and six mobile money features to understand whether any differences in behavioural traces were statistically significant. The results are illustrated in Fig. 4. On average those who moved to a poorer neighbourhood had lower entropy in the numbers called ($p < 0.05$), less money coming into their account overall ($p < 0.05$) as well as by means of putting “cash in” ($p < 0.05$).^8^ Notably, while the effect related to phone number entropy held on data *before* the person moved, the effect for ‘money in’ did not (see Fig. 5). This may indicate that moving to a poorer area negatively impacts the amount of money coming in to a migrant’s mobile money account, however, follow-up analyses would be required to assert causality.

### Prediction performance

The data derived from *before* migrating to Dar es Salaam was then used to train a predictive model. The goal was to investigate whether it was possible to predict which individuals were going to migrate to poorer and more deprived areas of Dar es Salaam—and if so, which features were indicative of such migration. The pipeline that achieved highest performance accuracy on unseen test data utilised a logistic regression classifier with 10 features selected, suggesting that linear relationships exits between the features and migrating to a deprived urban area.

**Figure 6:**
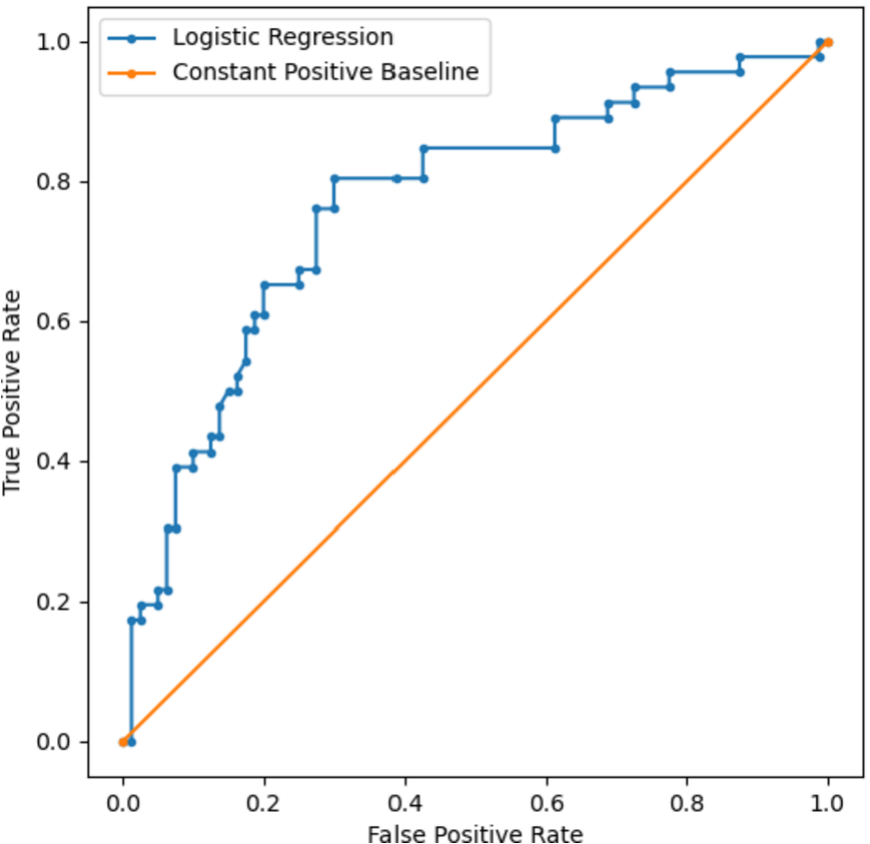
ROC curve for the logistic regression model compared to a constant positive baseline. The model had an Area Under the Curve (AUC) score of 0.76

The classification accuracy of the best model was 0.72, with a F1 score of 0.64, performing significantly better that a constant positive baseline ($\mathrm{F1} = 0.53$) and a random baseline ($\mathrm{F1} = 0.34$). A comparison of the ROC curve for the logistic model ($\mathrm{AUC} = 0.77$) compared to the constant positive baseline ($\mathrm{AUC} = 0.5$) can be viewed in Fig. 6. McNemar’s test of homogeneity [68] showed that the logistic regression model made significantly different errors, and has a different relative proportion of errors compared to the constant positive baseline ($p < 0.0001$).

**Figure 7:**
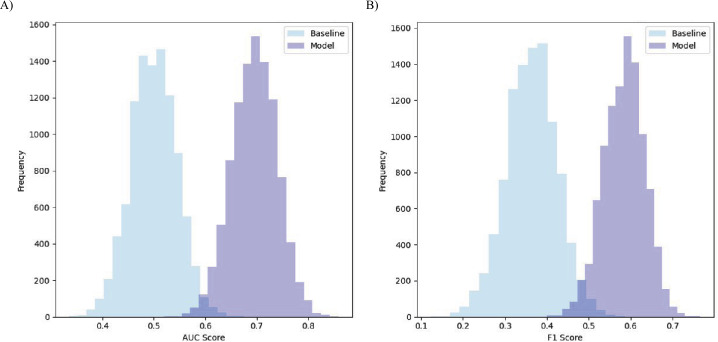
Distributions of bootstrapped scores for the logistic regression model compared to the random baseline

To further evidence that model performance was a statistically significant improvement over the baseline, resampling with replacement for $N = 10\text{,}000$ samples was conducted to produce a distribution of performance metrics for the random baseline^9^ and the model. Both the F1 score and the AUC scores for the model were significantly better ($p < 0.0001$) [69] than the scores for the baseline (see Fig. 7 for plots of the distributions).

**Figure 8:**
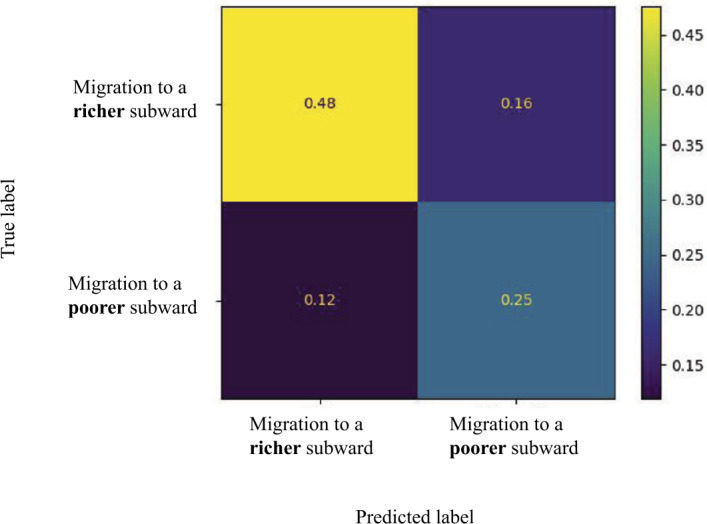
Confusion matrix (normalized to be between 0 and 1) for the best performing logistic regression model with an $\mathrm{F1}$ score of 0.65

The confusion matrix in Fig. 8 shows that the logistic regression model was slightly better at predicting those who moved to affluent subwards (0.75 accuracy), for which more data were available, compared to those who moved to more deprived subwards (0.67 accuracy).

### Model interpretation

Using optimized parameters obtained from model training (validated to ensure the best performing logistic regression model wouldn’t over-fit), the modelling pipeline was re-fit to the full dataset. This allowed use of all available data, increasing support in the interpretation of coefficient values when investigating which features were the best indicators of vulnerable migration. The regression coefficients of the optimally fitted logistic regressor can be found in Table 2.

**Table 2:** The logistic regression model’s *b* coefficients

Rank	Feature	Coef.
1	Deprivation of subward most phoned	0.56
2	Average amount (TZS) spent per day	−0.39
3	Home region’s education level^∗^	−0.22
4	Deprivation of ward most phoned	0.18
5	Home region: Pwani	0.15
6	Percentage of calls to Dar es Salaam	0.14
7	Home region: Mara	0.13
8	Home region: Shinyanga	−0.13
9	Entropy of phone numbers called	−0.12
10	Home region: Manyara	0.11

Note: For the binary outcome variable 1 = Migration to poorer subwards, 0 = Migration to more affluent subwards. TZS = Tanzanian Shillings. Features are ordered by the absolute coefficient value, with the most predictive feature first. All features were calculated on data before the individual migrated to Dar es Salaam. ^∗^From the 2014 UN Human Development Report for Tanzania. The variable measures the contribution of education to deprivation in the region, where a higher number represents poorer education.

The strongest predictor in the model proved to be the affluence ranking of the *subward* a migrant most commonly called in Dar es Salaam prior to moving. Also highly selected (rank 4) was the affluence ranking of the *ward* most often called. Both these features, which are very similar,^10^ suggest that people are migrating to areas where they already have social contacts (or at least areas with a similar level of deprivation). Intuitively, when a migrant is in contact with someone from a deprived area prior to migration, they are more likely to end up in such a setting themselves. The percentage of the calls an individual makes to Dar es Salaam ahead of migration, as well as the entropy of phone numbers called (where lower entropy predicted migrating to poorer subwards), were also useful predictors. This suggests that it is not just where social contacts reside, but the strength and size of social networks which predict migration to vulnerable urban areas. These findings are considered in more detail in the Discussion.

The second most important predictor emerging was the average amount of money spent by an individual (using mobile money) prior to migrating. The less people spent, the more likely they were to end up in a poorer subward—likely indicating lower disposable income, but also potentially reflecting saving strategies in preparation for relocation.

From the socio-demographic variables, the educational level of the region a migrant was moving from proved most influential. Crucially, the poorer the education in an originating region, the more likely individuals were to migrate to an area of high deprivation/unemployment in Dar es Salaam. Moreover, migrating from certain regions, in particular, Pwani (directly outside of Dar es Salaam) and Mara (in the agricultural far north), were indicative of migrants moving to deprived subwards; in contrast migrating from Shinyanga predicted moving to a more affluent area. Notably, many the variables left unselected by the model corresponded to source region’s ‘push factors’, such as the human development indices and variables depicting regional poverty.

## Discussion

This study leveraged pseudonymized commercial call records and mobile money data to study urban domestic migration to Dar es Salaam, Tanzania’s largest city. New migrants are recognized as a potentially vulnerable population at a greater risk of exploitation than local inhabitants [33]. This research sought to explore the impact of migrating to a more deprived neighbourhood in the city, where individuals are expected to be most vulnerable. The goals were to understand the behavioural and financial differences visible in digital traces when migrating to more deprived urban subwards, compared to more affluent, via statistical analysis of social and economic measures after the person moved; and to better understand what factors *predict* migration to a deprived urban area using a machine learning approach.

Statistical tests showed that people who migrated to a poorer area had less money coming into their mobile money account prior to migration. This intuitive reflection of lower affluence, however, was only observed after moving, *and not before*. This suggests that the deprivation level of *where* someone moves to in Dar es Salaam could be a determining factor in how much mobile money they acquire after migration, reflecting the importance of location within a city for employment and economic prospects. Migrants to poorer areas also displayed lower entropy in the phone numbers they called, both before and after moving, suggesting a smaller, less varied support network—potentially indicating an increased risk of social isolation.

Using only data available prior to migration, we then demonstrated the ability to predict whether a migrant will end up in one of the poorer or richer subwards in Dar es Salaam (achieving 72% classification accuracy). Classifiers suggest that not only are there social and economic differences between two broad migrant groups before they move, but that it is possible to characterise potential vulnerability of migrants prior to migration to a new city. Given the absence of accurate survey data in a city, projected to grow 100% from 6.7 million in 2020 to 13.4 million in 2035 [71], such models allow not only for future projections, but the potential to aid intervention strategies—and help better plan the targeting of resources to those who are most in need. The prediction of vulnerable migration also has the potential to be improved further (if explanations/interpretability is no longer the goal) by heavily weighting the vulnerable population and using more powerful ‘black box’ algorithms.

By analysing the coefficients of the best performing model, we found that features indicating the deprivation level of where a migrant’s social contacts spend time in Dar, as well as the amount of calls made to the city ahead of moving, to be useful positive predictors of an individual moving to a deprived area. Spending less mobile money prior to moving also predicted vulnerable migration, as did a lower level of education. Importantly perhaps, a poor educational index was the only regional human development factor which was significantly predictive in the model. This finding quantitatively corroborates previous theoretical research on education and migration [72] and highlights that opportunities, emerging as a result of mass urbanisation in low-income nations, are not equal for all migrants. In terms of policy, this result further emphasises the need to highlight educational requirements of many higher paid jobs that, while attracting migrants to cities, may remain unavailable to many of them.

Other expected push factors such as the region’s poverty, gender inequality, human development indices, female representation in parliament, healthcare, vaccinations, healthy births, and population demographics did not prove to be useful indicators of a migrant’s final situation. This may be a result of a high variance within regions, but also serves to highlight the importance of education above other development indicators in promoting opportunities and success for migrating individuals.

An individual making a high percentage of calls to (anywhere in) Dar es Salaam before moving was found to be predictive of migrating to a vulnerable area. Furthermore, the predictive model and *t*-tests showed that migrants to poorer areas were in contact with a smaller variety/number of individuals (low entropy of numbers called) both before and after moving. It is likely that those who migrate to the more economically deprived areas have smaller social networks, and consequently could be influenced by a narrower set of principles/ideologies (yet this hypothesis requires further analysis). Such individuals may have less opportunities (both before and after moving to Dar es Salaam) due to being less well connected, and potentially more socially isolated, despite the frequency and dependence on those connections. Conversely, those with a wider social network, are more likely to be subject to a greater variety of employment opportunities. Interestingly, the overall *number* of calls made by a migrant before moving was not a predictive feature—rather it is the variety of *different* people they call which predicts migration to a more affluent area of Dar es Salaam. These empirical findings corroborate literature on the importance of social networks in understanding successful migration [59].

Those who had contacts in deprived areas of Dar es Salaam were also more likely to end up in deprived areas themselves. A potential interpretation is that migrants temporarily reside with (i.e. on the same premises) or nearby prior social contacts whilst establishing themselves in a new locale. As available input data only spans a single calendar year, we can not determine whether these findings hold over a longer period of time. However, it is not possible to rule out alternative theories; that mobile phone contact to deprived areas of Dar es Salaam prior to migration is indicative of qualitatively noted ills, such as coercive recruitment strategies into exploitative situations [2]. Further investigations would be needed to understand whether these reflect genuine social contacts in Dar es Salaam, or whether these findings cast further concern over exploitative urban migration in Tanzania.

Spending less money per day before moving was predictive of migration to a poorer subward of Dar es Salaam. This could be because such individuals are simply migrating from a region where mobile money infrastructure is less prevalent. However, given the prevalence of mobile money across Tanzania, and ubiquity of mobile phone usage at all levels of society, it is likely that differences in pre-migration financial strategies exist. The group of individuals who migrate to the poorer areas of Dar es Salaam have greater likelihood of migration without employment/future income already established. Such individuals are also more likely to use their mobile money account as a means to save money before moving [19, 22]. Identifying different financial strategies for urban migration using mobile money data combined with surveys would be a productive future avenue of research.

Three different source regions were found to be highly indicative of people moving to poorer areas in Dar es Salaam (Pwani, Mara, and Manyara), whilst coming from one region (Shinyanga) predicted migration to a more affluent neighbourhood. The Pwani region is the area directly surrounding Dar es Salaam, whilst the sparse Manyara and Mara regions lie north west towards Kenya and the shores of lake Victoria. According to figures in [29], there is little correlation between these findings and the poverty and human development indices for these regions—supporting the fact that our model didn’t select other push factors as predictors. It is likely that more subtle historical, tribal and cultural drivers explain these regional differences [73]. Such regional findings are useful for making local policy decisions that impact uncontrolled and unplanned migration from across the country.

The above observations, elicited from individuals’ interactions with a mobile banking application and a cell network provider, contributes a new interface into the nature of migration in East Africa. While the quantitative insights produced are of most relevance to urban planners, social support agencies, policy makers, and others studying migration and vulnerability in Tanzania, we believe they also open the doors for a new conversation: Should mobile platform providers be doing more to support the communities who use their technologies, especially given the risks involved in migration, and the increased prevalence of migration being promoted by their use [16]?

### Data challenges and limitations

Despite the digital data traces used in this analysis offering new ways to study urban migration, there exists several limitations within the data. Firstly, the call detail records and mobile money data are from a single provider of several existing in Tanzania and Dar es Salaam. Despite this limitation, we note that (i) in urban populations mobile phone penetration has been reported to be close to 92% and (ii) the data provider had, at the time of data collection, a 28% market share and of these over 70% of the customers reported they only used one network [74]. To ensure the privacy of customers, no demographic information was obtained about the individuals or the sample as a whole. Without this demographic information, we can not statistically analyse how representative the data is of the general migrant population in Tanzania.^11^ Literature on mobile money demographics in Tanzania suggest that although mobile money is slightly more prevalent among households where consumption is above $2 a day, mobile money is used by more than a third of all households, and over a quarter of rural households [25]. Regretfully, migrants whose destination was the district of Temeke in Dar es Salaam were removed from the sample due to sparse network coverage, historically different network governance, and the less urban nature of the district. As care must be taken in the interpretation of the results, we recognize that the data cleansing and pre-processing steps could have unintentionally (and disproportionately) invisibilized already disadvantaged individuals (e.g. those with reduced cell network infrastructure).

Furthermore, we can only speak for differences in economic activity measured using mobile money. Some individuals, particularly those who have government jobs in Dar es Salaam, will additionally own a bank account at a more formal financial institution. We hypothesize that those who migrated to the most affluent areas are most likely to have a bank account in addition to mobile money. This, as well as the short time frame analysed after people migrated, may offer explanation as to why there weren’t stronger significant differences in mobile money accounts between those who moved to poorer versus more affluent areas. Finally, the data supplied to us consisted of a random sample of people from the mobile network provider’s full customer base. The method in which call records were extracted enforced that no information could be accessed on who people in the sample *received* calls from (unless they were also in the sample). This meant that we were restricted in our ability to study inter-network connectivity and the social networks that exist in such cross-market data.

## Conclusion

This study synthesized data on mobile phone calls, mobile money interactions, and survey responses to explore urban migration in Tanzania. We found that people who settled in poorer, more deprived neighbourhoods of Dar es Salaam had less money coming into their mobile money account after they moved, but not before. These migrants were also found to have a smaller and narrower social network, measured using phone call entropy. A machine learning model, built to predict which migrants will move to the poorer subwards in Dar es Salaam, found that features indicating the strength and location of migrants’ social connections in Dar es Salaam before they moved (‘pull factors’) to be most predictive, more so than traditional ‘push factors’ of the source region, such as poverty, with the exception of education. Poor education of the source region predicted migrants would move to a more deprived area in Dar es Salaam. Although limitations of the data are recognized, this study demonstrates the utility of using digital traces to study migration and vulnerability in low-income countries, where regular surveying proves a challenge. Moreover, prediction accuracy illustrates the potential for the data and methodology to be harnessed in application to automatically identify points of intervention, or where resources would be best targeted, to help prevent new urban migrants from entering into vulnerable circumstances. This work has also highlighted the discussions to be had with data owners on responsible and ethical interfacing into such data, in order to support research and interventions focused on the elimination of migrant exploitation.

## Supplementary Information

Below is the link to the electronic supplementary material.

The names, descriptions, and sources of all the features engineered and collated for this research (.csv file) (ZIP 80 kB)

## Data Availability

The raw mobile money and call detail records data is commercial and cannot be shared. However, the authors have made public the code and the modelling data (including the final list of filtered variables) used for this analysis, which can be viewed and downloaded from GitHub: https://github.com/Rosa-Lavelle-Hill/vulnerable-migration.

## Footnotes

1 Due to both individual and commercial privacy, the pseudonymized data used as part of this study is not publicly available, and was provided to us through a partnership with a mobile network operator with high market penetration in Tanzania. However, a link to the code used in this study along with the engineered feature dataset is available in the section Availability of Data and Material.

2 Comparisons corresponding to (ii) were recorded as a tie, and outcomes corresponding to (iii) were discarded and the judges were not asked about the subwards they were unfamiliar with again.

3 Note that we only examined data until the end of the year, and so we cannot determine how long people stayed in Dar es Salaam beyond 2014, hence the use of the phrase ‘semi-permanent’.

4 We note here that the cell phone data was from 2014, but the on the ground surveys were conducted in 2018. Despite the fast urbanisation, on balance (given the available literature [62, 63] and colleagues residing in Dar es Salaam’s experiences) it is believed that the survey still accurately depicts the relative affluence of neighbourhoods as they would have been roughly three and a half years prior.

5 If <10% of someone’s overall call volume went through the calculated mode ‘night tower’, instead the overall mode tower was used to prevent irregular night visits being used to identify a ‘home’ subward (this occurred for 6.48% of migrants in our sample).

6 The 50% cut-off was used to create a more balanced dataset, maximising the number of positive class labels in the training and test set. Available literature [64, 65], as well collaborators residing in Dar es Salaam’s experiences, also support a dichotomy between rich and poor neighbourhoods in the city.

7 Used only prior to logistic regression classifiers, as for both decision tree and random forest feature selection occurs intrinsically

8 When *p*-values were adjusted to control for increased false discovery rates due to multiple tests [67], they no longer reached the $p < 0.05$ threshold.

9 A normal distribution of F1 and AUC scores can not be produced for the constant positive baseline when using stratified *k*-fold validation (as was done in the modelling phase) to ensure each fold contains approximately the same percentage of samples of each target class as the complete data.

10 Due to the two features having a high correlation ($r=0.83$), and this having the potential to effect the interpretation of coefficients [70] a separate model was refit without the affluence of the ward feature. The results showed that the affluence of the subward coefficients increased, as expected, and the other coefficients remained stable.

11 The data providers user base is anecdotally reported as well distributed across the market.
